# Full-Field Indentation Damage Measurement Using Digital Image Correlation

**DOI:** 10.3390/ma10070774

**Published:** 2017-07-10

**Authors:** Elías López-Alba, Francisco A. Díaz-Garrido

**Affiliations:** Departamento de Ingeniería Mecánica y Minera, Campus las Lagunillas, Universidad de Jaén, 23071 Jaén, Spain; fdiaz@ujaen.es

**Keywords:** contact, indentation, damage, 3D digital image correlation

## Abstract

A novel approach based on full-field indentation measurements to characterize and quantify the effect of contact in thin plates is presented. The proposed method has been employed to evaluate the indentation damage generated in the presence of bending deformation, resulting from the contact between a thin plate and a rigid sphere. For this purpose, the 3D Digital Image Correlation (3D-DIC) technique has been adopted to quantify the out of plane displacements at the back face of the plate. Tests were conducted using aluminum thin plates and a rigid bearing sphere to evaluate the influence of the thickness and the material behavior during contact. Information provided by the 3D-DIC technique has been employed to perform an indirect measurement of the contact area during the loading and unloading path of the test. A symmetrical distribution in the contact damage region due to the symmetry of the indenter was always observed. In the case of aluminum plates, the presence of a high level of plasticity caused shearing deformation as the load increased. Results show the full-field contact damage area for different plates’ thicknesses at different loads. The contact damage region was bigger when the thickness of the specimen increased, and therefore, bending deformation was reduced. With the proposed approach, the elastic recovery at the contact location was quantified during the unloading, as well as the remaining permanent indentation damage after releasing the load. Results show the information obtained by full-field measurements at the contact location during the test, which implies a substantial improvement compared with pointwise techniques.

## 1. Introduction

The mechanical contact and the indentation damage experimented between two bodies under loading have been extensively studied and investigated in the past. The first study to develop a theory of the behavior of two elements in contact was provided by Hertz [1]. However, in many situations, the limits of Hertz’s theory are exceeded when a permanent indentation, once the yield strength of the material is exceeded, occurs during the experiment. Some efforts have been made to consider the effect of permanent indentation [2], even for the unloading path [3]. Early studies were focused on the contact analysis of the elastic/elastoplastic behavior of isotropic materials [4]. Decades after, the contact analysis in anisotropic and orthotropic materials was an important issue in the analysis of new materials [5,6,7]. However, when half space conditions are not achieved, bending stresses due to the indenter displacement are superimposed on the contact stress problem [8]. For a flexible target, the surface under contact will experience indentation and a force-deflection relationship due to the deformation of the target [9].

Many works have been conducted involving experiments using plates and a spherical indenter to validate analytical and numerical models [10,11,12]. In most of the studies, bending was avoided by assuming that the plate was rigidly supported or half-space conditions were achieved. In a real situation when the plate is loaded, the contact behavior depends on the plate thickness and its deflection [13]; therefore, the size of the contact damage will depend on this effect. Recently, Chen et al. [14] developed an analytical model that incorporated the influence of the specimen thickness to explain the effect of bending during contact. None of the reported methodologies were able to differentiate the displacements that were caused by bending and contacting [6,7,15]. Swanson and Rezaee [16] emphasized the importance of the depth of penetration and the size of the residual crater after unloading as a result of the material softening, concluding that the assumption of a half-space gave an underestimated stiffness value. Other authors [17] highlighted the importance of the contact area investigating the effect of impact velocity on the indentation produced. Thus, they employed a marker paint to measure, after the experiment, the maximum contact area during impact, finding some differences in the indentation measurement depending on the velocity test.

The mentioned research used a pointwise technique, which obtained the maximum indentation, but does not offer information regarding the complete region of interest. This paper presents a full-field experimental methodology to characterize the contact damage size evolution and indentation depth of thin plates in the presence of bending deformation. A better understanding of the region of interest could be achieved using this methodology, compared with the traditional pointwise techniques. A quasi-static contact experiment, using a rigid bearing sphere on aluminum plates, has been adopted to quantify the contact damage size and the maximum indentation depth occurring during the tests. In a conventional contact study, the indenter hides the area of interest presenting a limitation for its measurement during the experiment. In the present work, a full-field, optical technique, namely 3D Digital Image Correlation (3D-DIC), has been adopted to obtain information about the contact zone. Using the recorded information and knowing the indenter geometry, it was possible to apply a geometrical relationship obtaining information related to the hidden contact area. Preliminary studies using DIC to analyze the contact phenomenon were reported for other applications [18]; however, no research was found related to the determination of the evolution of contact damage area in real time. 

Experiments were performed using aluminum plates with thicknesses of 2, 3, 4, 5 and 6 mm. The contact damages and the bending deformation experienced variation depending on the stiffness of the specimen. The evolution of the contact damage for the loading and unloading path was analyzed under bending deformation. In the current paper, an alternative methodology based on the 3D-DIC technique has been developed and implemented to evaluate using the evolution of contact during indentation experiments. The proposed methodology provides full-field information from the rear face of the specimen where contact occurred, making it possible to evaluate the evolution of contact damage during the tests. By implementing this methodology, it has been possible to observe and evaluate the material elastic recovery and the generation of permanent damage of the specimens during experiments, showing the ability and potential of the proposed methodology.

## 2. Experimental Methodology

**Figure 1:**
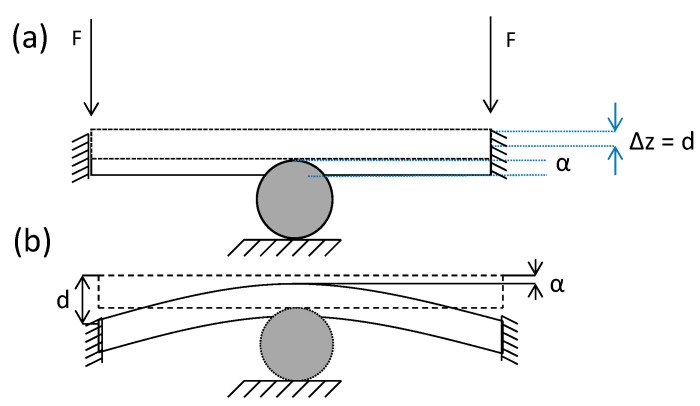
Mechanical contact between a plate and a sphere. (**a**) Schematic illustration showing the indentation and no bending produced in the plate; (**b**) indentation measurement principle during a contact experiment with the presence of bending.

In a mechanical contact between a rigid sphere and a plate, the contact damage is the crater generated by the sphere in the plate under loading conditions. Considering the sphere fixed and displacing the half space plate against the sphere, the indentation would be the z displacement experienced by the plate. We remark that the indentation α is defined as the thickness reduction experienced by the plate, considering a rigid sphere. Figure 1a shows a theoretical half space case when bending does not exists. The measured Δz displacement is the indentation α that occurred during contact. In this case, Δz is equal to the displacement of the plate d as a result of the movement of the plate against the indenter. When bending occurred (Figure 1b), measuring on the upper face of the plate, opposite of the contact area, will allocate the point with the minimum out of plane displacement that provides the indentation α for each loading step. From Figure 1b, it can be observed that maximum out of plane displacement, d, will occur at the edge of the plate, and it will provide the bending displacement. Thus, the total bending experienced by the plate will be the difference between d and α. In Figure 1b, an alternative measurement system would be necessary to quantify the indentation measurement.

**Figure 2:**
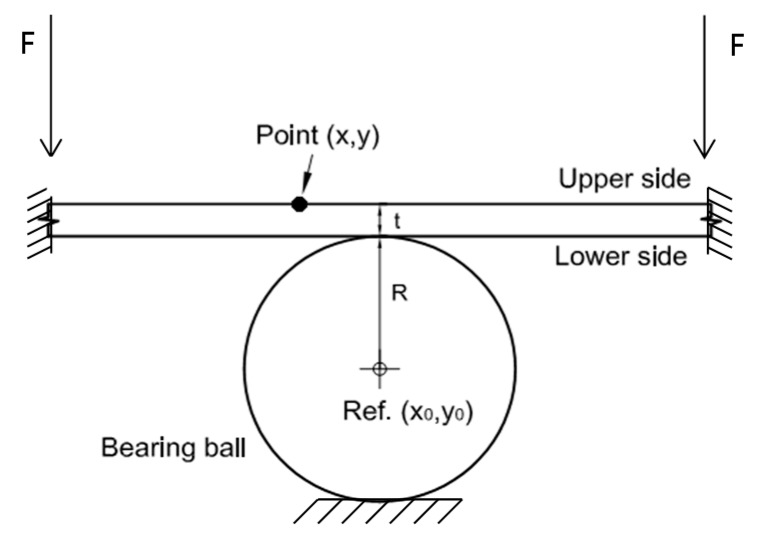
Original schematic of the bearing ball and the specimen.

One major problem in a contact experiment is that the contact area is hidden. The proposed methodology provides an indirect measurement of the contact area during the test. Therefore, only information is extracted from the upper face of the specimen when contact occurs. Full-field information is provided by 3D-DIC positioning the cameras focusing on the upper face of the specimen. By measuring the out of plane displacements provided by the 3D-DIC technique, it is possible to extract the contact area during the loading and unloading path of the test. Figure 2 shows a scheme of the original position of the plate and the bearing ball when contact starts. Every point P(x,y) experiences an out of plane displacement measured by 3D-DIC. It must be remarked that for the present analysis, it is assumed that the bearing ball has a very high stiffness, and consequently, it does not suffer any deformation during contact.

**Figure 3:**
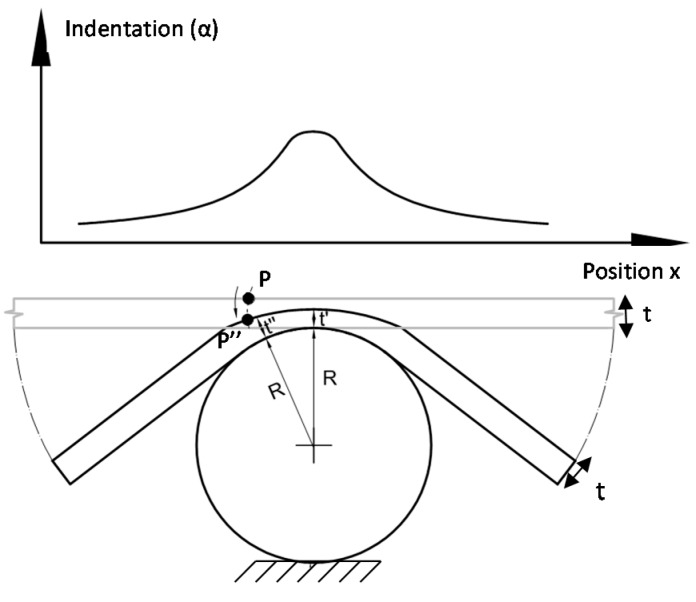
Schematic showing the contact behavior between a plate and a sphere. Detail of the damage generated due to contact.

Figure 3 shows the contact behavior between a plate and a sphere. When the load starts increasing, part of the sphere penetrates into the specimen, generating a contact damage area. The limit of this contact area is defined by the position of the sphere contour, where no contact with the plate exists. As the load starts increasing, the specimen thickness decreases at the contact region. This thickness reduction is not uniform, presenting deeper indentation at the center of the contact area (t’). The thickness (t’’) reduces towards the center of the contact area (t’), as is shown in Figure 3. Outside of the contact region, the thickness should be constant and equal to the original thickness of the specimen (t). Thus, every point P on the upper surface of the specimen experiences a displacement to a new position, P’’, on the deformed geometry, as shown in Figure 3. 

**Figure 4:**
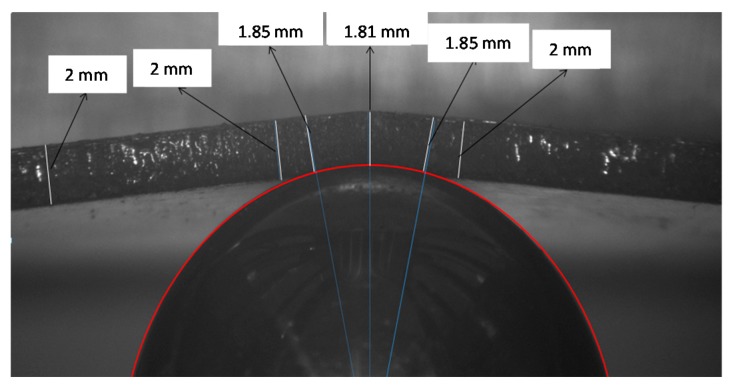
Experiment showing the contact area and the thickness reduction during the loading for a 2 mm-thick specimen and a sphere through the thickness.

An experiment was performed to validate the thickness reduction evolution, as was indicated in Figure 3. For that, the contact region of a sphere with a 2 mm-thick specimen under loading condition was studied, observing the contact damage through the thickness. Results shown in Figure 4 represent the thickness reduction in the contact region and the thickness constant value outside this area.

**Figure 5:**
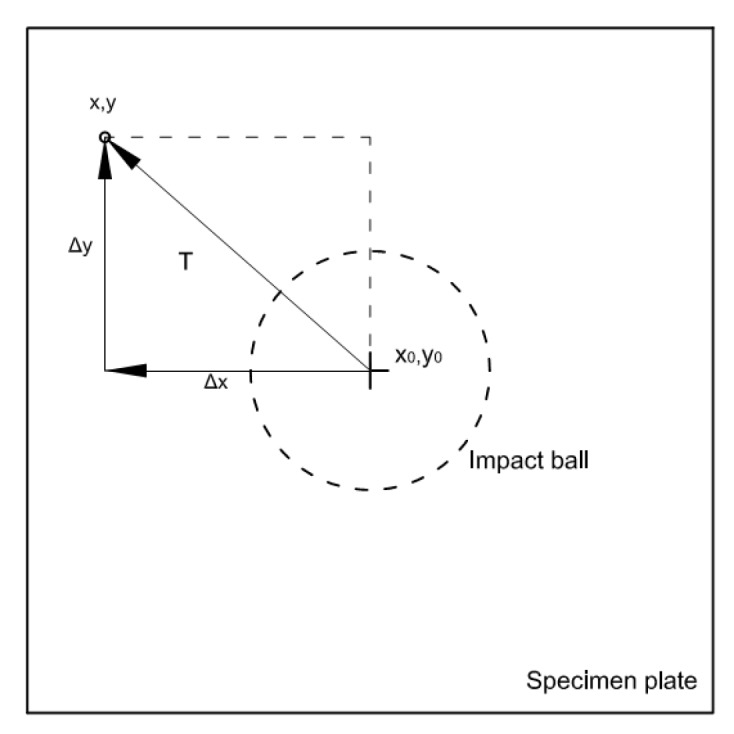
Definition of the point coordinates (x,y) at the upper specimen surface referring to the minimum out of plane displacement point coordinates (x_0_,y_0_).

Figure 5 illustrates the coordinate position on the plate. The position of any point at the upper surface of the specimen can be defined as a vector T with coordinates x and y referring to the point of maximum indentation (x_0_,y_0_), as shown in Figure 5. The components of the vector T are obtained from 3D-DIC results.

**Figure 6:**
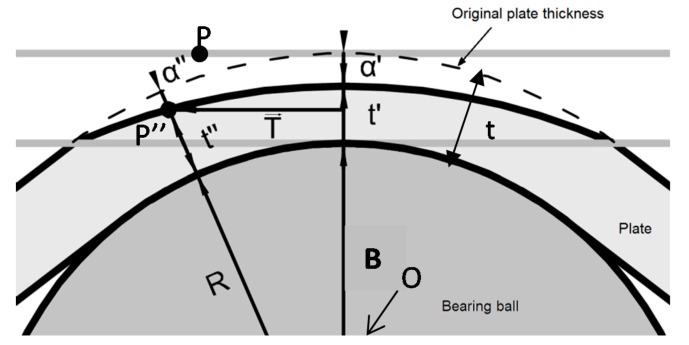
Sketch showing the adopted criteria to identify if a surface point is affected by thickness reduction due to contact.

Figure 6 shows how the position of a point P changes to P’’ when contact damage occurs. Point O represents the center of the sphere. If the radial distance from the center of the sphere OP’’ or R + t’’, is smaller than R + t, it means that the specimen has a thickness reduction. Therefore, it represents the contact damage region. At the limit of the contact region, the radial distance OP’’ is R + t (where R is the radius of the sphere).

Therefore, the geometrical position of OP’’ is defined as the sum of vectors T and B. The vector T is the projected distance on the reference plane xy shown in Figure 5, and B is P’’ distance in the thickness direction with respect to the sphere center and measured with 3D-DIC. Thus, from Figure 6, the following equations could be obtained:

B = R + t − α’’(1)

T = √ (x^2^ + y^2^) (2)

OP’’ = √ (B^2^ + T^2^) (3)

where R is the radius of the sphere, t the original plate thickness, α’’ the out of plane displacement experienced by point P after the load application and x and y are the coordinate of the point P’’. Thus, the condition to have contact damage would be:

OP’’ ≤ R + t(4)

where t is the original thickness of the plate and t’’ the specimen thickness on the contact region at the position of P’’.

Reorganizing Equations (4) and (5), it is achieved that where the sphere is in contact with the plate, contact damage will be present during the test.

OP’’ − R ≤ t(5)

**Figure 7:**
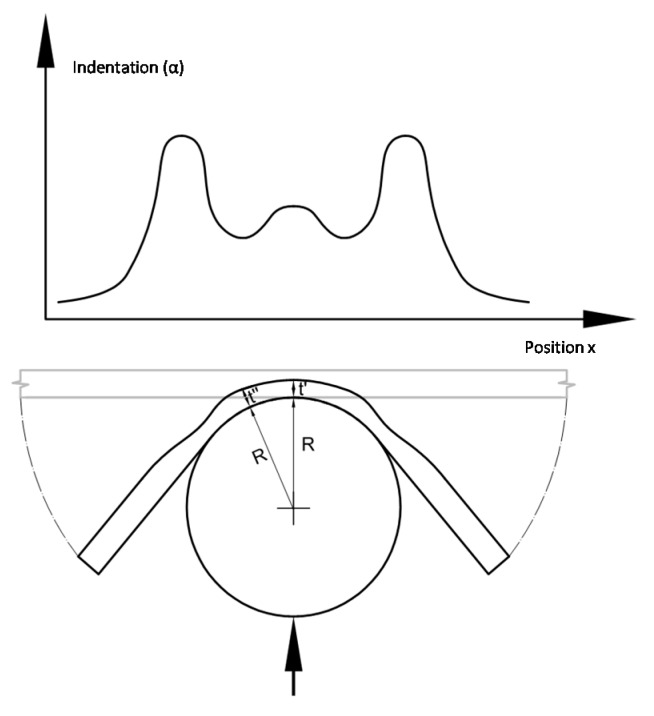
Scheme of the damage contact region when shear is predominant increasing the load applied.

If during the loading path, shear stress is predominant because higher loads are applied, it produces a higher deformation with a bigger thickness reduction in the region surrounding the center of the contact area, having a thickness distribution profile similar to that shown in Figure 7 [19].

Based on this methodology, a script was programmed into Matlab to post-process the images captured during the test. The geometry of the contact damage was obtained during the test to evaluate the thickness reduction.

## 3. Specimen Preparation and Experimental Setup

Specimens were manufactured from commercial AL-1050 H-14 (Dissa, Jaén, Spain, Table 1). The geometry adopted for experiments was square plates of 150 mm × 150 mm. The plates were flat, and no warps were observed over the surface.

The specimen surface was treated with white paint and subsequently a random speckle pattern over the white surface spraying a matt black paint. The random distribution is needs to apply 3D-DIC. The thickness of the applied coating was around 1 µm. Therefore, it was negligible compared with the magnitude of the measured displacements. The specimen was clamped 15 mm at each edge using a loading frame, leaving a free area of 120 mm^2^.

A calibrated stereoscopic system using two monochromatic CCD 5 Mpixels cameras (brand Allied Vision Technologies, model Stingray F-504B/C (Allied Vision Technologies GmbH, Stadtroda, Germany) was employed with two 23-mm focal length lenses (brand Schneider). Both cameras were synchronized and monitored from a laptop connected to a Data Acquisition system DAQ module. The specimen surface was properly illuminated. Finally, the displacements fields occurring at the specimen surface during loading and unloading were measured using a commercial software package (Vic-3D by Correlated Solutions Inc., West Columbia, SC, USA) [20].

**Figure 8:**
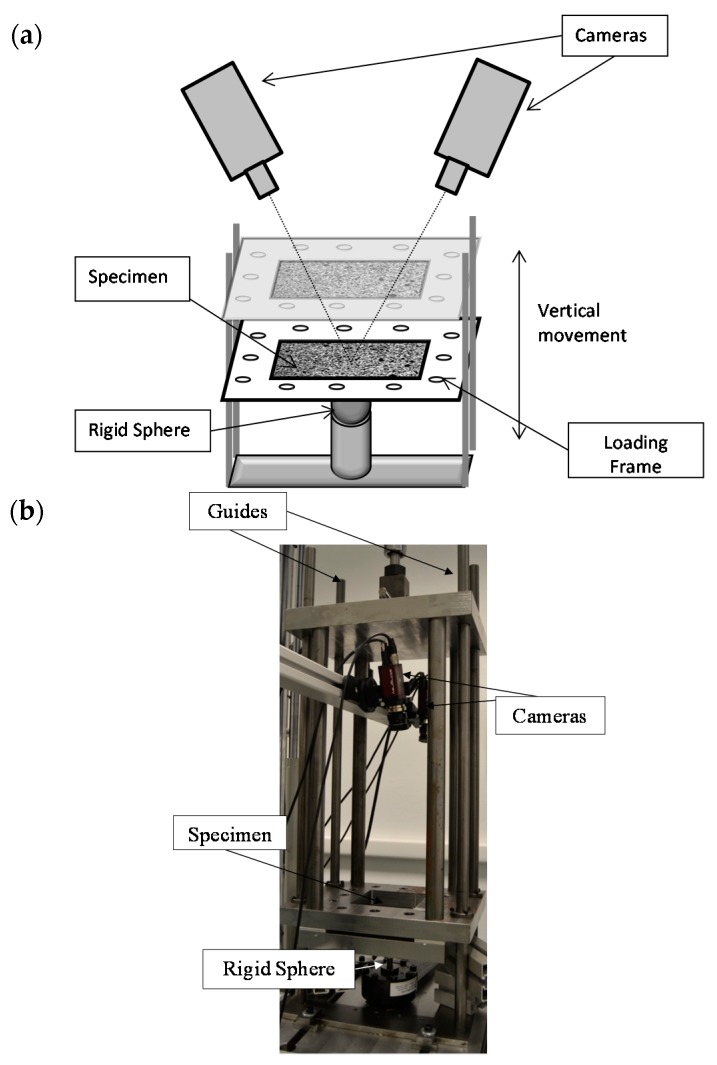
Experimental setup adopted. (**a**) Schematic illustration showing the setup to perform the test; (**b**) setup adopted for the experimental methodology proposed.

Tests were conducted using a MTS 370.02 servohydraulic machine (MTS Systems Corporation, Eden Prairie, MN, USA) with a maximum load capacity of 25 kN where a loading frame designed specifically for these test was clamped. The loading frame moved in the vertical direction (using the hydraulic actuator) through four guides to ensure a normal application of monotonic loading during experiments (Figure 8). Specimens were clamped into the bottom part of the loading frame. A 20-mm spherical indenter (steel ball bearing) was screwed to the load cell using a specially-designed adaptor, recording the load magnitude during experiments. The plate was previously lubricated, decreasing friction between the bodies.

Before starting the test, a 25-N preload was applied to guarantee the contact between the plate and the sphere (indenter). Subsequently, the test started, and a transistor-transistor logic pulse (TTL) was automatically generated to command the image acquisition from the DIC system. The hydraulic machine was generating +5 V TTL pulses to trigger the cameras in a synchronized way governing the optical system. Experiments were controlled using the displacement movement experienced by the hydraulic cylinder. For each displacement step, the load value, the cylinder displacement value and the image acquisition time were recorded. The damage area of the plate was indirectly quantified from displacements measured at the upper side of the contact surface.

The cylinder was moved with a displacement speed of 0.1 mm/s during loading and unloading. The maximum cylinder displacement was limited to 2 mm for aluminum specimens. Images were captured and post-processed to obtain the out of plane displacement field to infer the indentation and the contact damage area experienced by the specimen during each deformation step.

## 4. Validation Methodology

An initial experiment was conducted to evaluate the measurement uncertainty of the 3D-DIC technique for the analysis of contact problems using the proposed setup. In this case, the experiment was conducted without a specimen. Movement of the hydraulic cylinder was transferred to the movable part of the loading frame, and only a rigid body motion was measured without any load application. The actuator was moved 3 mm in six displacement steps of 0.5 mm. Hence, displacement readings provided by the hydraulic machine LVDT sensor were compared with those measured by 3D-DIC to obtain the measurement uncertainty of the technique. Thus, a statistical analysis was performed, and the mean, µ, and standard deviation, s, of the difference between 3D-DIC and the LVDT measurements were evaluated for each displacement step [21]. In general terms, the trend of the evaluated experimental results matched with the LVDT readings with a very low scatter. The average mean for the different displacements step was 0.0011 mm and the average standard deviation 0.0004 mm. Statistical calculations clearly show the high level of concordance.

In order to ensure that the setup, used in this work, satisfies the quasi-static contact law, two experiments were conducted to quantify its accuracy and repeatability. Indentation measurements were performed 10 times with the aid of a dial indicator and 10 times using 3D-DIC for a 2 mm-thick specimen. During the experiment, the hydraulic cylinder was moved 2 mm down (once the indenter touched the specimen) in steps of 0.1 mm at a speed of 0.1 mm/s. A dwell was programmed between load steps to trigger the cameras using a TTL pulse generated by the servohydraulic machine. The dial indicator, with an accuracy of 0.001 mm, was placed at the center of the sphere over the specimen.

**Figure 9:**
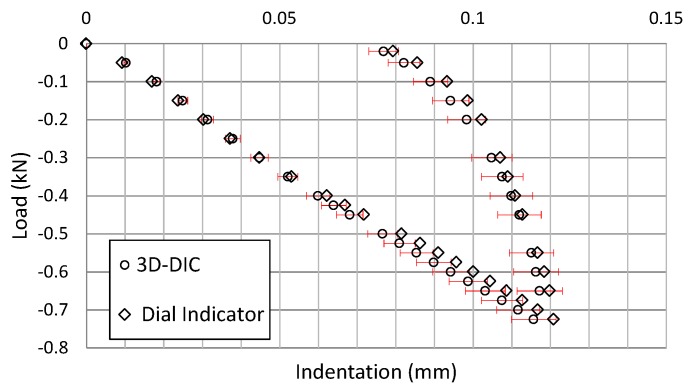
Results comparison using 3D Digital Image Correlation (3D-DIC) and the dial indicator.

Figure 9 shows a comparison of the results using both techniques. In all of the cases, differences were smaller than 6% and used as a reference to calculate the 6% dispersion bands of the 3D-DIC results. These differences in the indentation values can be attributed to the position of the dial indicator or to a small loss of perpendicularity of the dial indicator needle when the specimen deforms due to bending.

As has been presented, the repeatability of the maximum thickness reduction measurements (maximum indentation) has been achieved. Therefore, an agreement using two different experimental techniques has been demonstrated. The 3D-DIC technique provides the indentation measurement and the information related to the contact area size between the indenter and the specimen. In addition, the results obtained measuring with the full-field technique offer the information related to the thickness reduction in the contact damage region where the sphere was in contact with the plate. With the dial indicator, it is possible to measure only at one point, and the difficulties to measure are high in the presence of any gap in the setup. Moreover, it has been observed that results obtained using 3D-DIC showed less scattering than those obtained using a dial indicator. It can be concluded that the adopted setup provides a robust and repeatable procedure to satisfy the quasi-static contact law of the material in the contact damage region.

## 5. Results and Discussion

**Figure 10:**
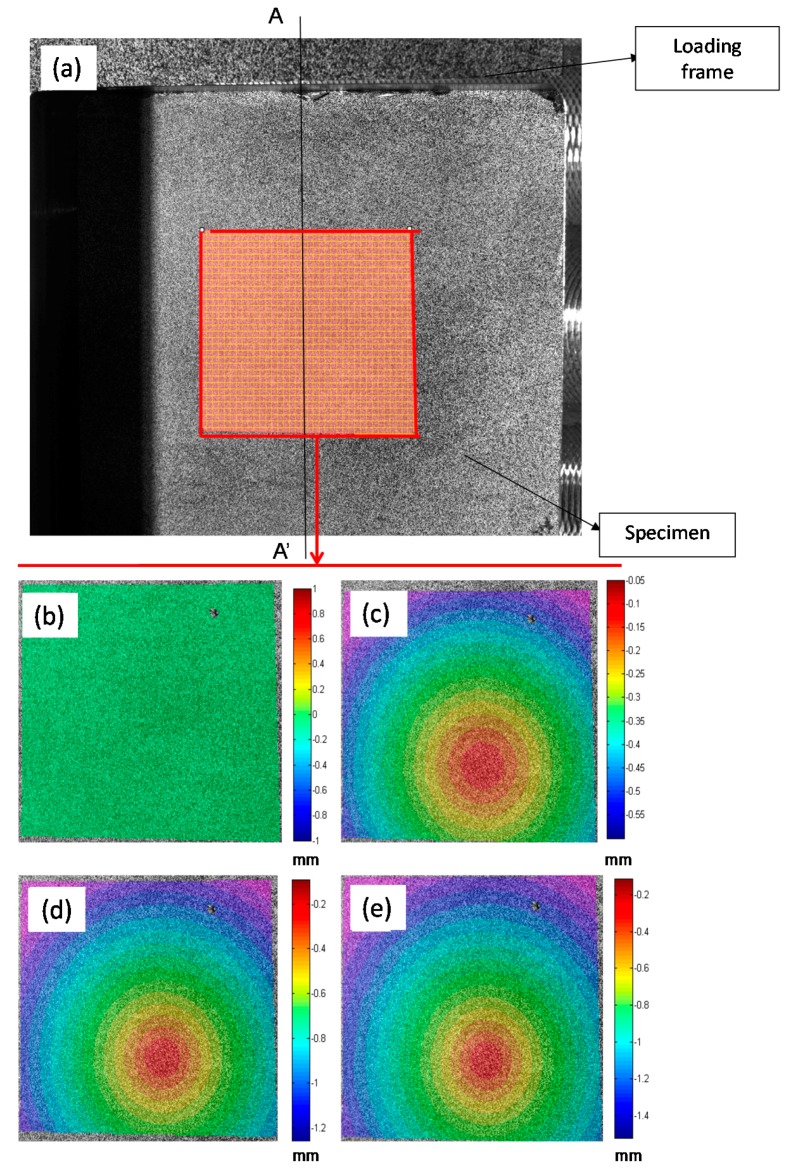
Region of interest for contact analysis. (**a**) Specimen image before starting a quasi-static test. Out of plane displacements measured in a 2 mm-thick specimen at the region of interest for different loads; (**b**) 0 mm to 0 N; (**c**) −0.05 mm to 320 N; (**d**) −0.096 mm to 611 N; (**e**) −0.111 mm to 731 N.

To determine the contact damage, images were captured during the test and subsequently post-processed using the 3D-DIC technique. A facet size of 25 × 25 pixels with two pixels of overlap was defined. Figure 10 shows an image captured by one of the cameras. The out of plane displacement profile along a line AA’ centered at the specimen is measured. In addition, Figure 10a, a detail of the full-field area where the sphere is in contact with the plate is shown. In Figure 10b–e, the out of plane displacement for different displacements steps is shown. 

**Figure 11:**
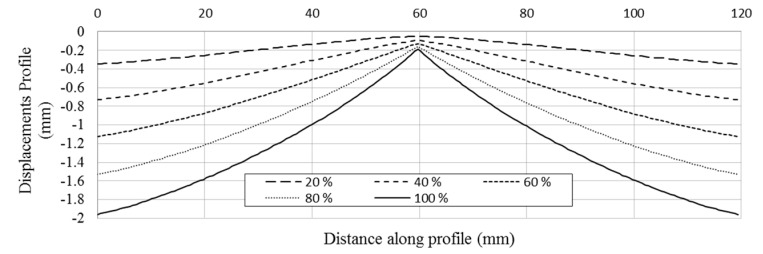
Evolution of the out of plane displacements along the profiles AA’ (defined in Figure 10a) at different displacement steps (in percentage) for a 3 mm-thick specimen, corresponding 100% to −2 mm cylinder displacement.

**Figure 12:**
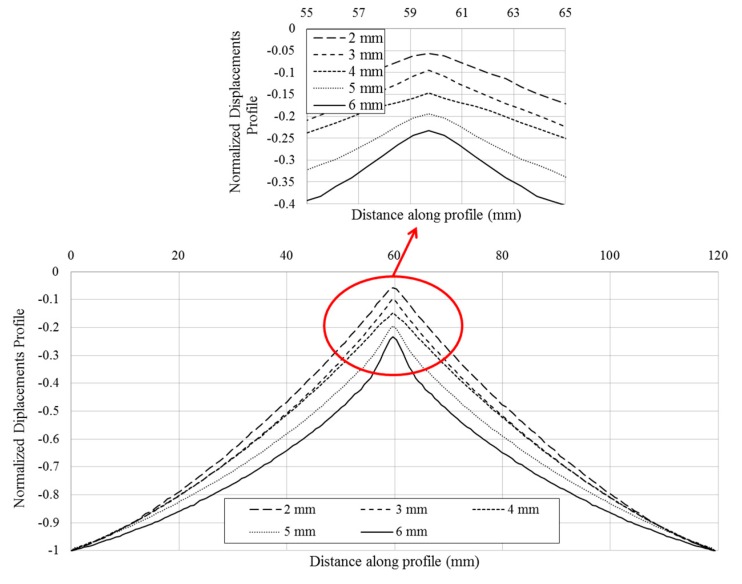
Normalized out of plane displacements profile by −2 mm cylinder movement versus distance along profile for different specimen thickness (2, 3, 4, 5 and 6 mm).

With the information extracted from the profile, the total movement of the hydraulic cylinder and the evolution of the out of plane displacements experienced by the specimen can be obtained. Figure 11 shows the out of plane deformation for a 3 mm-thick aluminum specimen at 20%, 40%, 60%, 80% and 100% of the cylinder displacement, respectively, corresponding to 100% to −2 mm. For the maximum cylinder displacement, the specimen showed a thickness reduction of 0.19 mm in the region where the sphere is contacting the plate. This minimum out of plane displacement evaluated represents the indentation generated by the sphere on the plate. To perform a profile comparison for the different deformation steps, Figure 12 shows the normalized out of plane displacement by −2 mm (maximum displacement of the cylinder) versus the specimen length for all of the thickness of the aluminum specimens tested. A zoom of the region of interest was shown. As for high thicknesses, the sphere penetrates deeply into the plate, resulting in greater contact damage.

From the presented results, it is concluded that there was a thickness reduction in the contact area. This reduction was largest at the center of the contact area (top of the sphere in contact with the plate). Thus, the indentation increased with the specimen thickness for the same cylinder displacement. This is attributed to the influence of the specimen thickness and supported by the fact that bending decreases when the specimen thickness increases. Thus, a stiffer specimen will experience more contact damage and less bending deformation than a lower stiffness specimen (thinner specimen). An example of this effect is presented in Figure 12 with a zoom where the minimum out of plane displacement occurred.

**Figure 13:**
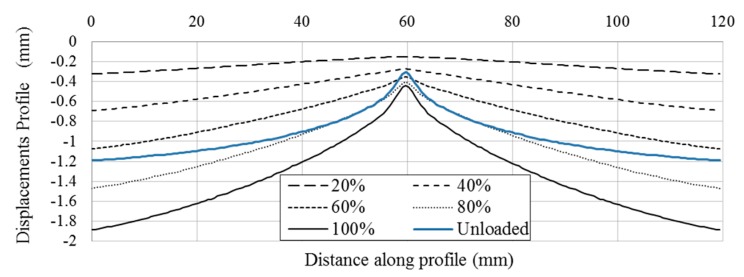
Evolution of the out of plane displacements along the profile AA’ (defined in Figure 10a) at different displacement steps (in percentage) during the loading and unloading final step for a 6 mm-thick specimen.

During the unloading path of the experiments, a recovery deformation was observed due to the elastic behavior of the material. Figure 13 shows a comparison between the loading steps and recovery deformation for the 6 mm-thick specimen. The absence of contact in the unloading path was identified as the moment at which the sphere lost contact with the plate, and consequently, no load was detected by the load cell. Thus, the measured out of plane displacements were associated with a permanent indentation in the contact region, as is shown in blue color. Therefore, it was possible to obtain a complete contact law for loading and unloading, as well as quantification of the bending deformation of the specimen. Similar results were obtained for different specimen thicknesses. Different recovery deformations were observed depending on the specimen stiffness.

**Figure 14:**
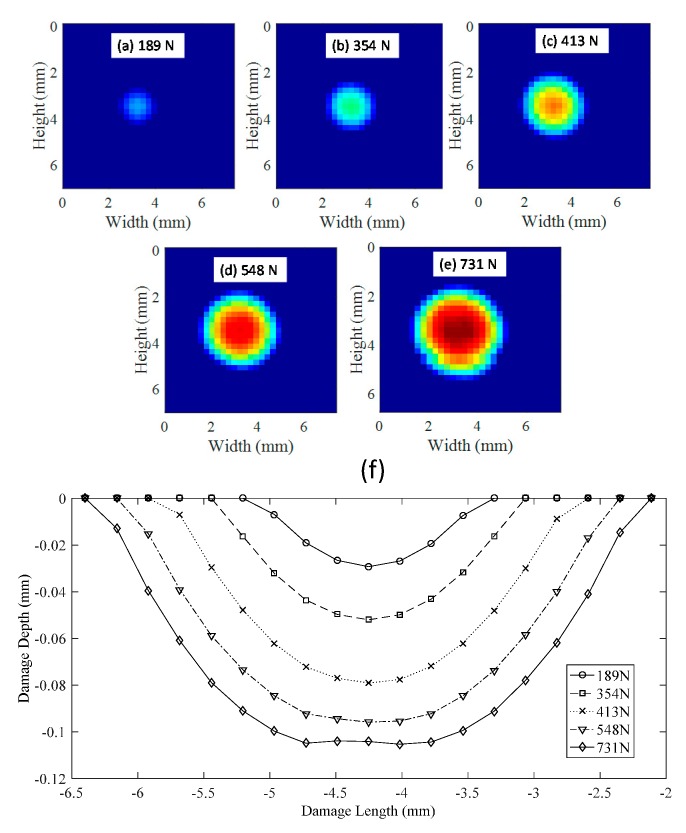
Damage geometry in the contact region during the unloading path for a 2 mm-thick specimen. (**a**–**e**) Geometry of the contact damage region for different displacement steps (20%, 40%, 60%, 80% and 100% cylinder displacement with a maximum of −2 mm) during the loading path for a 2 mm-thick specimen. (**f**) Profiles of the contact damage depth for the different displacements steps.

Figure 14 shows the results for a 2 mm-thick specimen. The geometry of the contact damage is shown at different displacement steps of 20%, 40%, 60%, 80% and 100% of the cylinder displacement. For each displacement, the applied load is shown, with a maximum load of 731 N measured. A symmetrical distribution of damage was experienced due to the symmetry of the indenter. At the maximum load, the initiation of shear deformation was observed. This effect happened because a flattening is present where maximum thickness reduction should occur. This event implies a higher thickness reduction surrounding the top point of the sphere in contact with the plate. However, this effect should be highlighted for other tests with higher contact damage produced. Figure 14a–e represents the thickness reduction and therefore the contact damage produced by the sphere in contact with the plate; a full-field view of the contact damage is shown. In Figure 14f, the contact damage profiles along line AA’, defined in Figure 10a, are shown for each displacement of the cylinder.

**Figure 15:**
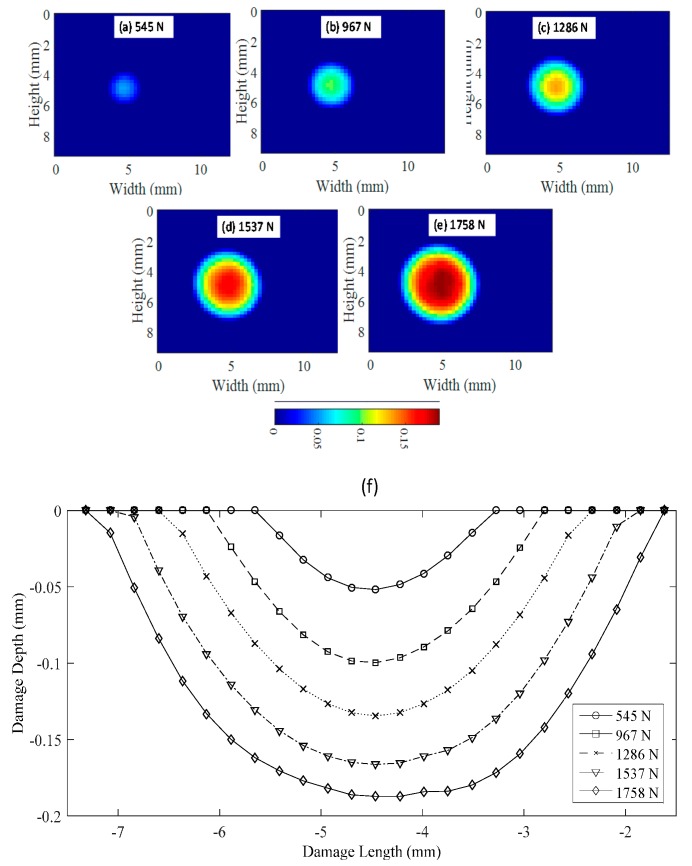
Damage geometry in the contact region during the unloading path for 3 mm thick specimen. (**a**–**e**) Geometry of the contact damage region for different displacement steps (20%, 40%, 60%, 80% and 100% cylinder displacement with a maximum of −2 mm) during the loading path for 3 mm thick specimen; (**f**) Profiles of the contact damage depth for the different displacements steps.

For a 3 mm-thick aluminum specimen, the results are shown in Figure 15. The maximum load applied to move the hydraulic cylinder at −2 mm was 1758 N. In this case, no shear nor flattening effects were observed in the contact region reaching a maximum indentation depth of −0.19 mm. Figure 15a–e represents the thickness reduction and therefore the contact damage size produced by the sphere in contact with the plate. Figure 14f illustrates the AA’ profiles (defined in Figure 10a) of the contact damage showing the depth damage for every step plotted. Similar results were obtained for thickness of 4 and 5 mm.

**Figure 16:**
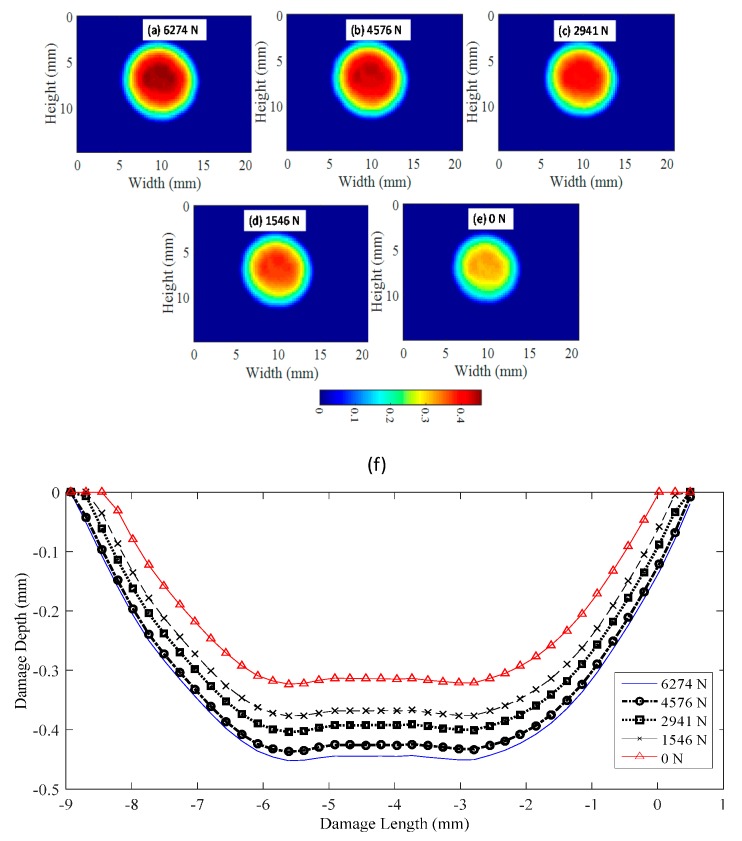
Damage geometry in the contact region during the unloading path for a 6 mm-thick specimen. (**a**) Maximum load reached to 100% of the cylinder displacement with a maximum of −2 mm; (**b**–**d**) Stages representing the elastic recovery during the unloading path; (**e**) Permanent damage in the plate; (**f**) Profiles of the contact damage depth for the different stages during the unloading path.

Figure 16 shows the geometry of the contact damage for a 6 mm-thick specimen. The maximum applied load for this test was 6274 N at −2 mm cylinder displacement, reaching a maximum indentation in the peak point of the bearing ball of −0.447 (blue color). It is observed that the specimen experienced more thickness reduction surrounding the peak point of the sphere in the contact area. This is attributed to the shear effect explained previously. Results of the geometrical contact damage were plotted for the maximum load state and for the unloading path. Once the maximum displacement was reached (blue color), the plate was unloaded, showing the elastic behavior of the contact damage. From this figure, the recovery deformation experienced by the specimen is observed when the load decreases, until non-contact between both bodies at 0 N (red color). This instant resulted in permanent contact damage. Similar results were obtained for different thicknesses. Figure 15a shows the maximum contact damage at maximum displacement of −2 mm and a load of 6274 N. Figure 15b–e illustrates the evolution of the contact damage during the unloading path until permanent contact damage was present at 0 N. Figure 16f shows the profiles AA’ (defined in Figure 10a) at the different steps indicated previously.

With a full-field view provided by 3D-DIC, it was possible to quantify the size of the contact damage. As was observed in Figure 14 and Figure 15 when the plate is thicker, the contact damage area is bigger and deeper. For 2 mm thick (Figure 14e), the maximum contact damage had a diameter of 3.83 mm. For 3 mm thick (Figure 15e), the maximum contact damage had a diameter of 5.25 mm. Figure 16a shows the contact damage at maximum load (−2 mm cylinder displacement) for a 6 mm-thick specimen; in this case, the contact damage had a diameter of 9.3 mm. Monitoring the test during the loading and unloading path, it was possible to evaluate the elastic recovery experienced by the material. Figure 16e shows the permanent contact damage of 8.12 mm in diameter. Thus, 12.69% of elastic recovery was experienced compared with the maximum load state.

In this study, a robust methodology has been presented that uses full-field displacement measurements to observe the evolution of contact damage with bending deformation. This offers a better understanding of the behavior of the plate during the loading and unloading path.

## 6. Conclusions

A full-field experimental methodology based on 3D-DIC has been presented to determine the real contact damage on metallic specimens. The adopted setup takes into account the influence of bending during contact experiments. Using a geometrical evaluation, it was possible to provide an indirect measurement of the contact region. The proposed methodology has been validated for the indentation depth using a pointwise conventional indentation measuring techniques. The differences between the indentation depths obtained with both techniques were less than 6%. To illustrate the proposed methodology, contact experiments using aluminum plates with 2, 3, 4, 5 and 6 mm thicknesses were conducted. Results from experiments made it possible to quantify the experimental contact damage geometry and the maximum indentation depth for different specimen’s thicknesses with the presence of bending deformation. It can be concluded that the contact damage area increases when the specimen thickness increases. When the bending deformation decreases, it was observed that the contact damage area was higher. The recovered elastic indentation during the unloading and the permanent contact damage created have been also successfully quantified. 

The adopted experimental methodology could potentially be used in future work to evaluate the specimen contact behavior at different loading rates, material stiffnesses, bending deformations of the specimens and the extrapolation of the methodology to dynamic events, such as impact.

## Figures and Tables

**Table 1:** AL-1050 H-14 properties.

Density (g/cm^3^)	Hardness Brinell (HB)	E (GPa)	ν	Yield Stress (MPa)
2.7	34	69	0.33	105
